# Sex‐differential associations of MC3R p.F45S with human metabolic profile

**DOI:** 10.1111/jne.70214

**Published:** 2026-06-16

**Authors:** Tanya C. Kaur, Andrew R. Wood, Gareth Hawkes, Robin N. Beaumont, Kartik Chundru, Michael N. Weedon, Hugh D. Piggins, Kate L. J. Ellacott

**Affiliations:** ^1^ School of Physiology, Pharmacology and Neuroscience University of Bristol Bristol UK; ^2^ Department of Clinical and Biomedical Sciences University of Exeter Medical School Exeter UK

**Keywords:** genetic variant, melanocortin, melanocortin‐3 receptor, metabolism, puberty

## Abstract

Obesity and related metabolic disorders represent major global health challenges, highlighting the importance of understanding the central regulation of energy homeostasis. The melanocortin system is a conserved circuitry governing food intake and other neuroendocrine processes. Within this system, the melanocortin‐3 receptor (MC3R) regulates energy and glucose balance, body composition, and linear growth in rodent models, with evidence of sexually dimorphic expression and function. Whether MC3R exerts similar sex‐specific effects in humans remains unclear, largely due to the rarity of loss‐of‐function (LoF) variants. We analysed data from the UK Biobank (UKB), a population‐based study comprising 500,000 individuals, to investigate the phenotypic consequences of the rare MC3R LoF variant rs143321797 (p.F45S). Using the largest UKB whole‐genome sequencing dataset to date, we performed comprehensive phenotypic analyses, including the first sex‐stratified assessment of this variant. Carriage of the MC3R p.F45S variant was associated with differences in adult stature and the timing of sexual maturation. Though the variant was not associated with increased risk of obesity or metabolic disease, carriers exhibited an altered adipose tissue distribution profile characterised by relatively greater subcutaneous fat deposition. Our findings independently validate the role of MC3R in human stature and sexual maturation and identify a previously unreported association with adipose tissue distribution. The absence of increased obesity or metabolic disease risk in MC3R p.F45S carriers of this study, together with a subcutaneous‐biased adipose distribution, suggests that MC3R may influence metabolic health through regulation of adipose tissue distribution rather than overall adiposity.

## 1 INTRODUCTION

The global rise in metabolic disorders such as obesity and Type 2 Diabetes places a substantial burden on healthcare systems and patients, highlighting the urgent need for effective therapeutic strategies. Since these conditions are driven by impaired energy regulation, improved understanding of the melanocortin system—a central circuitry that integrates signals across multiple brain regions to regulate energy intake, storage, and expenditure—is a vital research area.^1^, ^2^, ^3^ The melanocortin 3 receptor (MC3R) is a key component of this system, and functions as an energy rheostat.^4^ Within this role, MC3R directs calories towards energy‐storage (e.g., adipose tissue deposition) or energy‐demanding processes (e.g., growth and reproduction), in response to metabolic pressures to maintain the upper and lower bounds of energy homeostasis.^3^, ^4^ Given this regulatory function in energy partitioning, MC3R is a promising target for conditions characterised by chronic energy imbalance, like obesity or anorexia. This has been supported by recent findings in mice where co‐administration of an MC3R antagonist with tirzepatide—a dual GLP1R and GIPR agonist—was shown to significantly reduce food intake and body weight compared to either therapy alone.^5^

To effectively and safely target MC3R for the regulation of energy balance, further investigation is needed to translate and validate its physiological role from rodent models to humans. A previous genetic study that explored the role of MC3R in humans reported that carriers of MC3R loss‐of‐function (LoF) variants exhibit delayed puberty and reduced height, but only minimal changes in metabolic phenotype.^3^ The subtlety of this metabolic phenotype may reflect unexamined sex‐dependent effects. This is supported by rodent studies indicating pronounced sexual dimorphism in *Mc3r* expression across brain regions that regulate feeding and metabolism,^6^, ^7^ as well as sex‐specific differences in the feeding behaviour of MC3R‐deficient mice treated with anorexigenic agents.^5^, ^6^, ^7^ Together, these findings point to a sex‐dependent divergence in the melanocortin neural circuitry underlying metabolic regulation.

In humans, the sex‐specific functions of MC3R remain largely unexplored due to the rarity of LoF variants, yet clarifying these differences is essential for informing our understanding of the role of this receptor in human physiology, and subsequent development and application of MC3R‐targeted therapies. To address this, we utilised UK Biobank (UKB), a prospective population‐based study of participants in the UK,^8^ to investigate the phenotypic impact of an MC3R LoF variant, rs143321797 (p.F45S), on energy balance and metabolic disorders. With the recent availability of whole‐genome sequencing data from ~500,000 participants, we were able to examine the sex‐specific effects of this variant to determine whether its phenotypic associations differ between biological males and females.

## 2 METHODS

### 2.1 Cohort information

UKB is a population‐based, prospective study with over 500,000 participants recruited between 2006 and 2010. Participants were aged between 40 and 69 years at recruitment, with a sex distribution of approximately 54% female and 46% male.^8^ Prior to UKB‐based analysis, participants were stratified by broad genetic ancestry, based on principal components analysis and subsequent supervised machine learning approaches.^9^ All analyses were performed using unrelated individuals classified as of European genetic ancestry (377, 455 participants: 45% male, 55% female). UKB protocols were approved by the National Research Ethics Service Committee. This study was conducted under UK Biobank project 103,356.

### 2.2 UKB WGS data processing and variant selection

Annotation of the whole genome sequencing (WGS) dataset with the Ensembl Variant Effect Predictor (VEP v110)^10^ identified 4588 variants in ENST00000243911.

Variants for phenotypic testing underwent consecutive rounds of filtering. Firstly, we screened for variants exhibiting both a SIFT (v6.2.1) and PolyPhen (v2.2.3) score between 0–0.05 (predicted deleterious) and 0.98–1 (predicted probably damaging), respectively. Of the 106 variants matching these criteria, nine overlapped with human MC3R variants reported in the literature at the time of conducting this search (Tables S1 and S2). Of these nine variants, five have displayed a documented loss of in vitro cAMP signalling under site‐directed mutagenesis, which is used as a proxy for LoF^3^, ^11^, ^12^: p.F45S (rs143321797), p.L53R (rs61736060), p.D121Y (rs368205448), p.I146N (rs74315393), p.G240W (rs776955610).

Due to low allele frequencies preventing variant‐specific analysis, only p.F45S was taken forward. This variant had a minor allele frequency of 0.0675% and all carriers (510 total: 278 female, 232 male) were heterozygous. A phenome‐wide association study (PheWAS) was also conducted to preliminarily screen for associations of variants across endocrine/metabolic phenotypes (470 k, v5), but none reached statistical significance after Bonferroni correction for multiple testing (Bonferroni corrected *p* value <0.05).

### 2.3 Phenotype selection

Phenotypic outcomes were selected from UKB records based on relevance to puberty timing, anthropometric traits, dietary intake, markers of metabolism, and metabolic disorders. Variables were used as provided by UKB, except for those that required additional processing, as described below.

The fertile period was calculated as the difference between age at menopause [Field ID: 3581] and age at menarche [Field ID: 2714]. Variables involving fat and/or lean mass combined Dual‐Energy X‐ray Absorptiometry (DEXA) and bioelectrical impedance analysis (BIA) measurements, the latter of which were converted to DEXA‐equivalent estimates using validated prediction equations^13^; this permitted a greater sample size. Lean mass index incorporates an adjustment for height (lean mass / height^2^). All measurements pertaining to dietary intake excluded those with self‐reported atypical diets [Field ID: 100020]. Dietary intake variables were converted to kilocalories (kcal) using standard energy conversions and expressed as percentages of total energy intake. Energy balance was a measure of energy expended, which comprised basal metabolic rate (BMR), physical activity, and the thermic effect of food (TEF), divided by energy intake [Field ID: 26002]: BMR was calculated using the Mifflin‐St Jeor equation with the required candidate information^14^; energy used for physical activity was calculated by converting the metabolic equivalent of task (MET) candidate information to kcal/day [Field IDs: 22037, 22038, 22039]^15^, ^16^; TEF was calculated as 10% of total energy intake^17^ following conversion to kcal. All first recorded instances were taken. Age at diagnosis for obesity, non‐insulin dependent diabetes mellitus, and insulin dependent diabetes mellitus were calculated as the difference between date of birth and date of respective diagnosis.

### 2.4 Statistical analysis

All statistical analyses were performed using the Posit R Workbench on the UKB's Research Analysis Platform available through DNAnexus. Linear and logistic regression models were adjusted for genetic sex (where applicable) [Field ID: 31], age at assessment centre [Field ID: 21003], and principal components (PCs) [Field ID: 22009, PCs 1–40] as potential confounding variables. Association analyses involving proteomic variables were further corrected for UKB recruitment centre [Field ID: 54], WGS batch [Field ID: 32053], fasting time [Field ID: 74], and hour of blood draw [Field ID: 3166].^18^ A variant‐by‐sex interaction term was also included (where applicable) to test for sex‐specific effects of p.F45S. All dependent variables were analysed with both the full dataset and a dataset that excluded values beyond ±4 standard deviations, which served as the criterion for outlier removal. Where appropriate, phenotypic values were either log‐transformed or inverse‐normalised to improve model fit (Tables S3–S5). Multiple testing was controlled using a Bonferroni correction, where each *p*‐value was multiplied by the number of tests performed for that dependent variable (*p*_adjusted_ = *p* × *N*). Variables were considered statistically significant if the adjusted *p*‐value was <0.05.

## 3 RESULTS

### 3.1 MC3R p.F45S delays puberty onset and impacts adult stature

**FIGURE 1:**
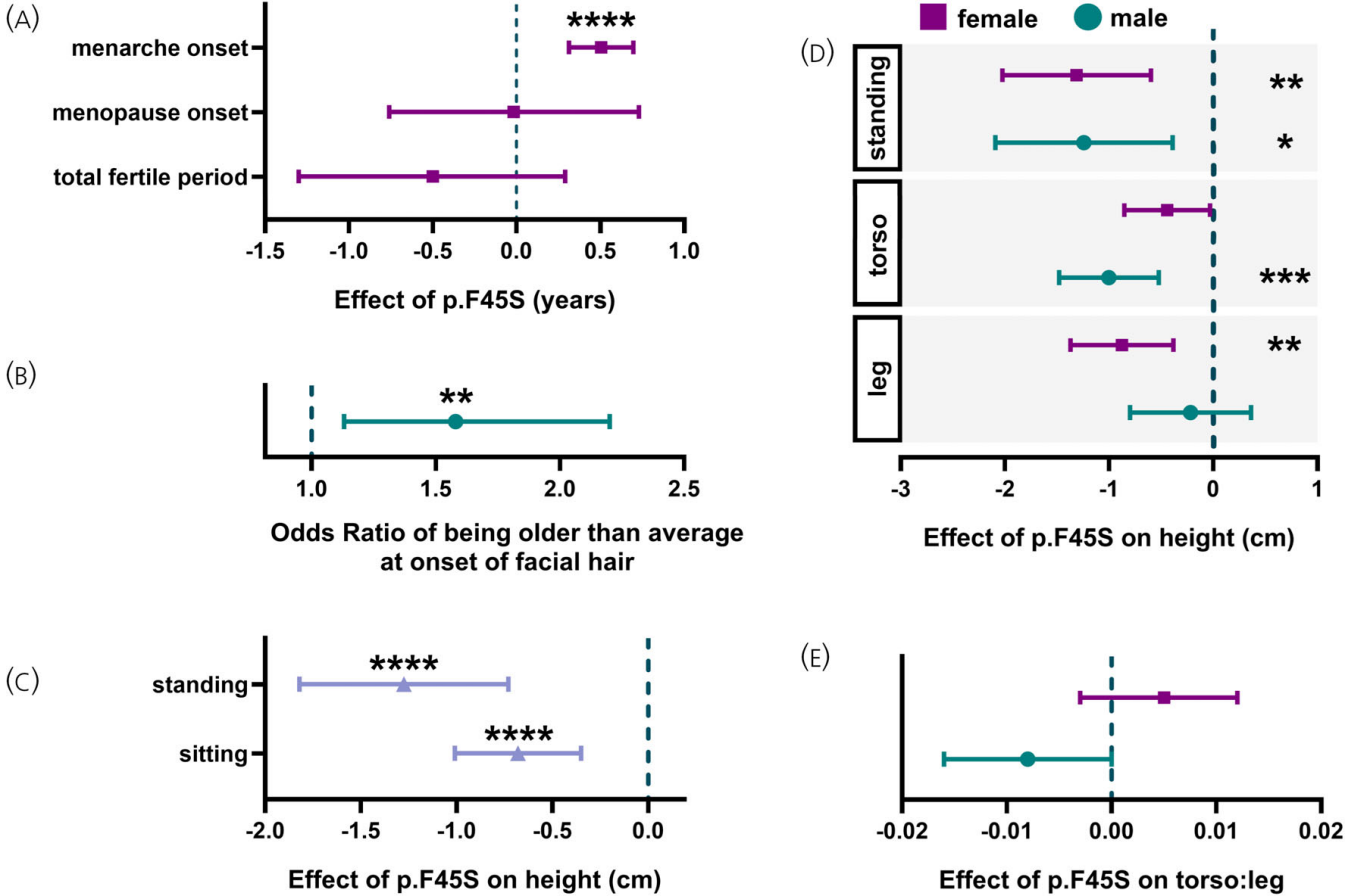
MC3R p.F45S affects puberty onset and linear growth. Forest plots depict the effect of MC3R p.F45S on (A) the age at menarche onset, menopause onset and total fertile period; (B) the odds of older‐than‐average age at onset of facial hair; (C) standing and sitting height in this UK Biobank cohort. The cohort was separated by genetic sex, and associations plotted for (D) different height parameters and (E) the torso‐to‐leg ratio. All plots display beta, or odds ratio, ± 95% CI. Colours and symbols indicate the sex cohort: Females only (purple squares), males only (green circles), or combined (grey triangle). Dashed lines indicate a null effect. Asterisks indicate significant Bonferroni‐corrected *p*‐value results from linear, or logistic, regressions: *p < 0.05; **p < 0.01; ***p < 0.001; ****p < 0.0001. N, beta values, odds ratio, standard error, and *p* values, for all variables are listed in Table S3.

Association studies employing UKB and other large‐scale human datasets have established a link between MC3R haploinsufficiency and reduced linear growth as well as delayed pubertal timing.^3^, ^11^, ^19^, ^20^ We first set out to verify whether these associations persisted with the LoF p.F45S variant in our European ancestry subset of the UKB. We observed a significant association of p.F45S with a delayed onset of menarche by 6.05 months (95% CIs: 3.74, 8.36 months; *p* = 5.78 × 10^−7^), and additionally report that the onset of menopause (beta = −0.017 years; 95% CIs: −0.76, 0.73 years; *p* = 0.99) and total fertile period (beta = −0.51 years; 95% CIs: −1.3, 0.29 years; *p* = 0.43) remained unaffected (Figure 1A). In males, we found that those carrying the p.F45S variant have 1.58× higher odds of being above average age at the commencement of facial hair growth (95% CIs: 1.13, 2.2; *p* = 0.006) (Figure 1B). In regard to adult stature, variant carriers exhibited significant decreases in both sitting height (beta = −0.7 cm; 95% CIs: −1.01, −0.39 cm; *p* = 7 × 10^−4^) and standing height (beta = −1.28 cm; 95% CIs: −1.82, −0.73 cm; *p* = 3.02 × 10^−5^) (Figure 1C). Together, these findings replicate and extend those of Lam et al.,^3^ strengthening the evidence that MC3R contributes to the regulation of growth and sexual maturation within humans.

To address the reported sex‐dependent differences in MC3R expression and function,^6^, ^7^, ^21^, ^22^, ^23^ we further explored the effect of the p.F45S variant on adult stature within males and females. Both sexes displayed significantly reduced standing heights; however, the proportions of this reduction differed: after correction for multiple testing, torso length contributed to height reduction within the male cohort (beta = −1 cm; 95% CIs: −1.48, −0.52 cm; *p* = 0.0002), whereas reduced leg length was the key contributor within the female cohort (beta = −0.88 cm; 95% CIs: −1.38, −0.38 cm; *p* = 0.003) (Figure 1D). Further examination of the torso‐to‐leg ratio revealed a nominal decrease (*p* < 0.05 prior to correction for multiple testing) within the male cohort only (Figure 1E).

To determine whether these phenotypic effects were significantly different between sexes, we conducted an interaction analysis including a variant‐by‐sex term. This revealed a nominal interaction effect for the torso‐to‐leg ratio only. Therefore, we conclude that overall stature is reduced in both male and female heterozygote carriers of p.F45S, with a potential for proportional differences between sexes.

To explore the underlying biology affecting this difference in stature, circulating levels of insulin‐like growth factor 1 (IGF1), and the proteomic expression levels of IGF1 receptor and IGF binding proteins (IGFBP) 1–4, 6 and 7 were investigated. Of these, only IGFBP6 was decreased, though this did not remain significant after correction for multiple testing (Table S4).

### 3.2 Sex‐specific associations of p.F45S with parameters relating to energy balance

The observed association of MC3R deficiency with reduced adult stature and delayed pubertal timing highlights its role in coordinating energy availability with developmental demands. This regulatory function in systemic homeostasis is reflected in MC3R knockout (KO) mice, which exhibit a markedly increased fat‐to‐lean mass ratio despite reports of either hypophagia or no change in ad libitum food intake, and no alteration in respiratory exchange ratio.^4^, ^24^, ^25^ MC3R's role in energy rheostasis becomes observable under metabolic challenges, as evidenced by the greater weight loss of MC3R‐KO mice during caloric restriction and their more pronounced weight gain on a high‐fat diet compared with wild‐type controls.^4^ Collectively, these findings illustrate disrupted nutrient partitioning and impaired metabolic adaptability in the absence of functional MC3R. To investigate whether this disrupted metabolic homeostasis extends to humans, we analysed a range of anthropometric, dietary, and metabolic parameters in carriers of the p.F45S variant.

**FIGURE 2:**
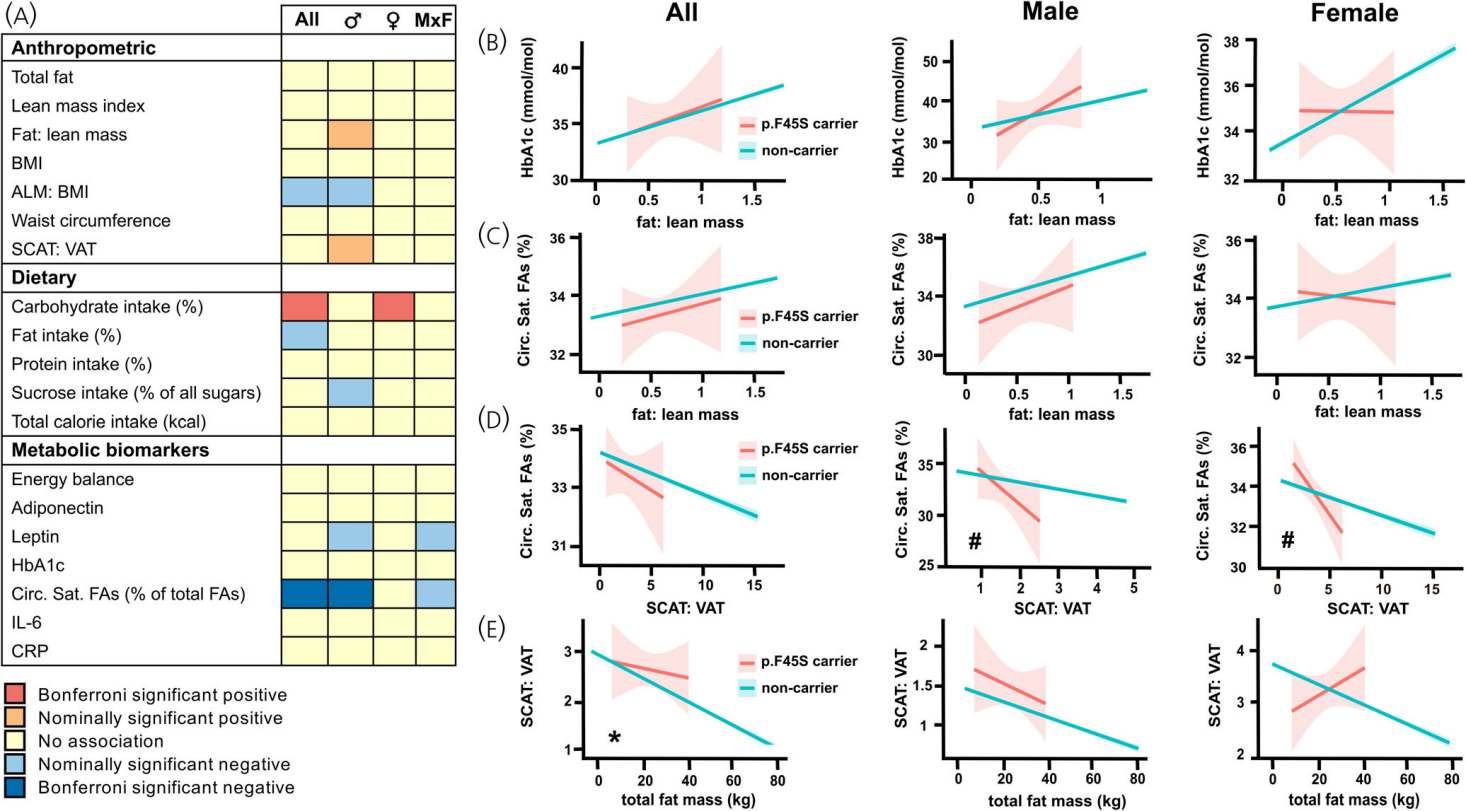
Roles of MC3R p.F45S in nutrient partitioning variables by sex. (A) Heatmap showing the associations of MC3R p.F45S with the listed variables, analysed in all participants, within genetic sex, and between genetic sex (MxF column). Associations were plotted between (B) fat‐to‐lean mass ratio and glycated haemoglobin levels, (C) fat‐to‐lean mass ratio and circulating saturated fatty acids, (D) subcutaneous‐to‐visceral fat ratio and circulating saturated fatty acids, and e) total fat mass and the partitioning between subcutaneous and visceral fat, to compare carriers and non‐carriers of MC3R p.F45S. Plots depict mean ± SE, and colours indicate carrier status: Heterozygote for p.F45S (orange); non‐carrier (green). Differences were assessed using a linear mixed model: *p < 0.05 for a main effect of the variant; # nominal *p* < 0.05 for a main effect of the variant. N, beta values, standard errors, *p* values, and correlation data are listed in Tables S5 and S6. ALM, appendicular lean mass; BMI, body mass index; Circ. Sat. FAs, circulating saturated fatty acids; CRP, C‐reactive protein; HbA1c, glycated haemoglobin; IL‐6, interlukin‐6; SCAT, subcutaneous adipose tissue; VAT, visceral adipose tissue.

Overall, our analyses revealed that male and female carriers of p.F45S exhibited a nominal decrease in their appendicular lean mass‐to‐body mass index (ALM:BMI) ratio (a noted measure of sarcopenia^26^). Though there was no statistically significant difference in total calorie intake based on carrier status, reported carbohydrate intake among carriers was higher (beta = 3.4%; 95% CI = 1%, 5.8%; *p* = 0.03), and levels of circulating saturated fatty acids were lower (beta = −0.3%; 95% CI = 0.95%, 0.51%; *p* = 0.02). There were no detected differences in the ratio of fat‐to‐lean mass, adipose distribution, changes in adipokines, glycated haemoglobin (HbA1c), and inflammatory markers (Figure 2A).

Sex‐stratified analysis revealed an altered profile in males, where carriers exhibited a nominally significant increase in their fat‐to‐lean mass ratio, with adipose tissue distribution skewed towards subcutaneous rather than visceral accumulation. There was no reported difference in total caloric intake, and only nominal decreases in circulating leptin and reported sucrose intake were detected, whereas circulating saturated fatty acids were significantly reduced (beta = −0.55%; 95% CI = −0.88%, −0.21%; *p* = 0.008). Collectively, these findings suggest a potentially metabolically protective phenotype associated with the p.F45S variant in males. In contrast, the female cohort showed no change in body composition or metabolic biomarkers (Figure 2A). Female carriers did, however, report a significant increase in carbohydrate intake (beta = 5%; 95% CI = 2%, 7.9%; *p* = 0.007), but no other differences were identified in our analyses. Variant‐by‐sex interaction analyses provided nominal evidence that male carriers of p.F45S exhibited lower leptin and circulating saturated fatty acid levels compared to female carriers (unadjusted *p* = 0.026 and 0.029, respectively).

Exploring the interplay of variables, rather than examining them in isolation, provides a more nuanced understanding of complex biological systems. This integrative approach captures the dynamic relationships and potential mechanisms that may be obscured when variables are considered independently. Therefore, to assess potential changes in relative metabolic health, we modelled the interaction between body composition measures and blood glucose control (HbA1c) and circulating saturated fatty acids. This analysis revealed that there was no difference between carriers and non‐carriers on either HbA1c levels or circulating saturated fatty acids relative to their fat‐to‐lean mass ratio (Figure 2B,C). HbA1c levels relative to total fat mass also showed no detectable differences (Figure S1a).

Given the distinct metabolic roles of adipose tissue depots, we then examined fat distribution patterns. Visceral adipose tissue (VAT) exhibits greater lipolytic activity and contributes to insulin resistance and systemic inflammation, whereas subcutaneous adipose tissue (SCAT) is generally regarded as a metabolically protective storage depot.^27^ In both males and females, carriers of the p.F45S variant with a higher SCAT‐to‐VAT ratio showed no difference in HbA1c levels (Figure S1b) but exhibited a nominal association with lower circulating saturated fatty acid levels, indicative of a more favourable lipid profile (Figure 2D). Further probing of adipose partitioning revealed that with increasing fat mass, variant carriers also significantly accumulated subcutaneous rather than visceral fat (Figure 2E). This disproportionate partitioning suggests an altered regulation of adipose tissue deposition in p.F45S carriers, where subcutaneous adipose storage is favoured. Interaction analyses did not reveal any evidence of sex‐dependent effects of the variant for these associations.

### 3.3 MC3R p.F45S neither protects nor confers risk towards metabolic disorders

**FIGURE 3:**
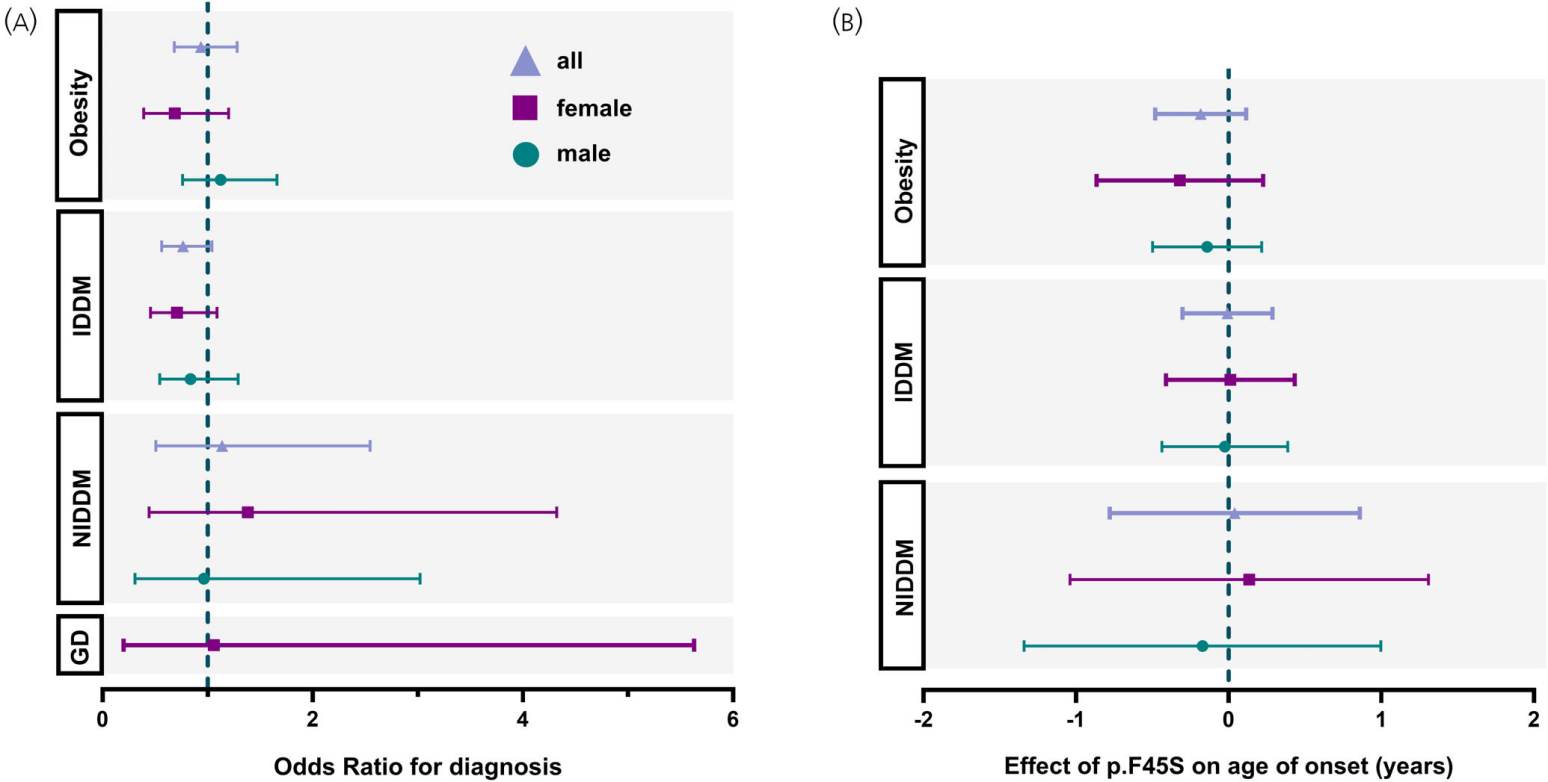
Association of MC3R p.F45S with metabolic disorders. Forest plots depict the effect of MC3R p.F45S on (A) the odds ratio of diagnosis, or (B) the age at diagnosis, for the tested metabolic disorders. Odds ratio, or beta, ± 95% CI are shown. Dashed line indicates null effect. Colours and symbols indicate the population sample analysed: Both males and females (grey triangles), females only (purple squares), males only (green circles). N, beta values, standard errors, and *p* values are listed in Table S5. GD, gestational diabetes; IDDM, Insulin dependent diabetes mellitus; NIDDM, non‐insulin dependent diabetes mellitus.

The nature of altered nutrient partitioning towards fat mass in mouse MC3R KO models often results in a mild obesity phenotype.^24^, ^28^ The susceptibility to obesity—particularly early‐onset obesity—in human carriers of MC3R variants remains inconclusive.^29^ An evaluation of p.F45S in a smaller German cohort reported that this variant was carried only by individuals with severe obesity,^19^ and homozygous LoF MC3R carriers present with a phenotype of severe obesity as well as Type 2 Diabetes,^3^ whereas recent findings across three large consanguineous cohorts report no excess of obesity in individuals with homozygous MC3R LoF mutations.^20^ In the UKB cohort, we found that the p.F45S variant confers no change in the likelihood of diagnosis, or onset of, obesity, insulin‐dependent diabetes mellitus, non‐insulin dependent diabetes mellitus, or gestational diabetes (Figure 3A,B).

## 4 DISCUSSION

Using WGS data from the UKB, our findings independently confirm previously reported roles of MC3R in regulating human sexual maturation and linear growth.^3^, ^20^ We found no effect on menopause onset or total fertile period in female carriers of p.F45S, indicating that MC3R's influence on reproductive transitions may largely be confined to the timing of sexual maturation. Puberty represents a critical window for linear growth, and our extended analysis of adult stature revealed that p.F45S affects body proportions differently within males and females, reflecting sex‐specific differences in skeletal development during this period. As disruptions in pubertal progression often reflect insufficient energy availability, this reinforces the role of MC3R in nutrient sensing and allocation to energy‐demanding physiological processes.^3^, ^30^

Similar to rodent studies, our analysis in a large human cohort highlights subtle disruptions in systemic metabolic phenotype among variant carriers. Importantly, we show that the p.F45S variant differentially affects circulating levels of saturated fatty acids and leptin between males and females (Figure 2A). Notably, for the same given SCAT: VAT ratio, there was a nominal decrease in the percentage of circulating saturated fatty acids in carriers compared to non‐carriers (Figure 2D). The significant decrease in overall circulating saturated fatty acids observed in male carriers may be linked to findings from MC3R KO models. In these models, impaired fatty acid oxidation has been associated with increased expression of CD36,^31^ a protein that facilitates the uptake of long‐chain free fatty acids, and decreased hormone‐sensitive lipase,^32^ which is responsible for mobilising stored fat.

We are the first to describe a difference in the relative deposition of adipose tissue, where MC3R p.F45S carriers have a significantly higher proportion of subcutaneous compared to visceral adipose, relative to total fat mass (Figure 2E). This presents another potential mechanism for the reduction in circulating fatty acids, as preferential uptake by SCAT has been reported.^27^ The difference in SCAT: VAT ratio may also underlie the differences in leptin levels seen between male and female MC3R p.F45S variant carriers, as there are reported differences in leptin secretion between these adipose depots, with SCAT having a relatively higher contribution to circulating leptin levels than VAT.^33^, ^34^, ^35^, ^36^ However, because these published data predict that relatively higher SCAT would be expected to be associated with higher circulating leptin, further investigation would be needed to understand mechanisms underlying the relatively lower leptin levels in biologically male MC3R p.F45S variant carriers, which may be related to differences in adipocyte function.

Our analyses suggest that the high SCAT: VAT ratio observed in MC3R p.F45S variant carriers is also associated with better metabolic outcomes, such as a lower risk of hepatic insulin resistance, lower HbA1c levels and a reduced inflammatory profile.^27^ Such a metabolically protective profile is supported by our reported lack of a predisposition to metabolic disorders (Figure 3), and by HbA1c levels that remain comparable to wild‐type individuals even as the fat‐to‐lean mass ratio increases (Figure 2A). In our study, sex‐dependent effects in metabolic phenotypes were either not detected or did not remain significant after post‐hoc correction. It is possible that, although biological male and female variant carriers exhibit similar physiological responses under baseline conditions, the underlying neural mechanisms are sex‐specific, with divergences becoming evident only under physiological stressors. This has been demonstrated by the distinct behavioural phenotypes observed in male and female MC3R KO mice when subjected to stress‐induced anorexia,^6^ but remain to be mechanistically explored in humans.

In mice, the importance of MC3R for energy rheostasis becomes evident during homeostatic challenges such as energy deficits and surpluses. For example, rodents without MC3R function exhibit greater weight gain than wild‐type counterparts in an orexigenic environment, exemplified by a high‐fat diet.^4^, ^37^ To explore this in human p.F45S carriers, a cohort of participants with a self‐reported typical dietary intake of more than 35% fat^38^ was created; however, the insufficient number of carriers in this cohort precluded further analysis. This remains a caveat in our reported phenotypes too, as p.F45S is a low frequency variant, so subtle effect sizes may not be detected. Further, we note that all carriers in these analyses were heterozygous, while phenotypes have been shown to be more pronounced in homozygous individuals.^3^

## 5 CONCLUSIONS

Overall, these findings verify MC3R's role in human linear growth and sexual maturation previously reported.^3^, ^20^ We further demonstrate that the metabolic effects of disrupted MC3R function on baseline phenotype are broadly conserved between rodents and humans, supporting the translational utility of mouse models for metabolic research. Multivariable analyses further reveal a more favourable adipose distribution profile in MC3R p.F45S carriers, where subcutaneous deposition is preferential. After adjusting for multiple testing, our analyses did not detect any statistically significant sex‐dependent effects of the p.F45S variant. However, rodent studies have demonstrated sex‐specific phenotypes under metabolic challenge, a context that could not be assessed in the present analysis but is worthy of exploration in future studies.

## AUTHOR CONTRIBUTIONS

**Robin N. Beaumont:** Data curation. **Hugh D. Piggins:** Supervision; writing – review and editing; funding acquisition; conceptualization. **Andrew R. Wood:** Data curation; supervision; methodology; resources; writing – review and editing; funding acquisition; conceptualization. **Kate L. J. Ellacott:** Supervision; writing – review and editing; conceptualization; funding acquisition. **Tanya C. Kaur:** Formal analysis; investigation; methodology; visualization; writing – original draft; conceptualization. **Gareth Hawkes:** Data curation. **Kartik Chundru:** Data curation. **Michael N. Weedon:** Data curation.

## FUNDING INFORMATION

Tanya C. Kaur was supported by a BBSRC‐funded South‐West Biosciences Doctoral Training Partnership (BB/T008741/1). Andrew R. Wood was supported by the Academy of Medical Sciences/the Wellcome Trust/the Government Department of Business, Energy and Industrial Strategy/the British Heart Foundation/Diabetes UK Springboard Award [SBF006\1134]. Gareth Hawkes is supported by the Medical Research Council grant UKRI327. Robin N. Beaumont and Michael N. Weedon are supported by Medical Research Council grant MR/Y003748/1. The research utilised data from the UK Biobank resource carried out under UK Biobank application number 103356. UK Biobank protocols were approved by the National Research Ethics Service Committee.

## CONFLICT OF INTEREST STATEMENT

The authors have no conflicts of interest to disclose.

## ETHICS STATEMENT

The research utilised data from the UK Biobank resource carried out under UK Biobank application number 103356. UK Biobank protocols were approved by the National Research Ethics Service Committee.

## Supporting information


**FIGURE S1.** MC3R p.F45S associations of HbA1c with fat mass variables. Associations were plotted between (A) total fat mass and glycated haemoglobin levels and (B) subcutaneous‐to‐visceral fat mass ratio and glycated haemoglobin levels, to compare carriers and non‐carriers of MC3R p.F45S within and between genetic sexes. Plots depict mean ± SE, and colours indicate carrier status: heterozygote for p.F45S (orange); non‐carrier (green). Differences were assessed using a linear mixed model, but there were no significant differences. N, beta values, standard errors, p values, and correlation data are listed in Table S6. HbA1c, glycated haemoglobin; SCAT, subcutaneous adipose tissue; VAT, visceral adipose tissue.

**TABLE S2.** Shortlisted variables predicted to cause loss of function (with SIFT and PolyPhen filtering).
**TABLE S3.** MxF refers to the value reported for the variant: sex interaction.
**TABLE S4.** Summary of IGF1, IGF1R, and IGFBP associations.
**TABLE S5.** Shaded areas denote omitted analyses, either due to excessive skewness in data distributions, or because transformation was unnecessary for already normal distributions or logistic analysis, or it was unnecessary due to a sex‐specific condition.
**TABLE S6.** Summary of inter‐variable correlations.

## Data Availability

The data that support the findings of this study are available from the corresponding author upon reasonable request.
