# Supplementary figures and images for: Sex‐differential associations of MC3R p.F45S with human metabolic profile
Source: J Neuroendocrinol. 2026 Jun 16;38(6):e70214. doi: 10.1111/jne.70214 (PMC13272279; doi:10.1111/jne.70214)

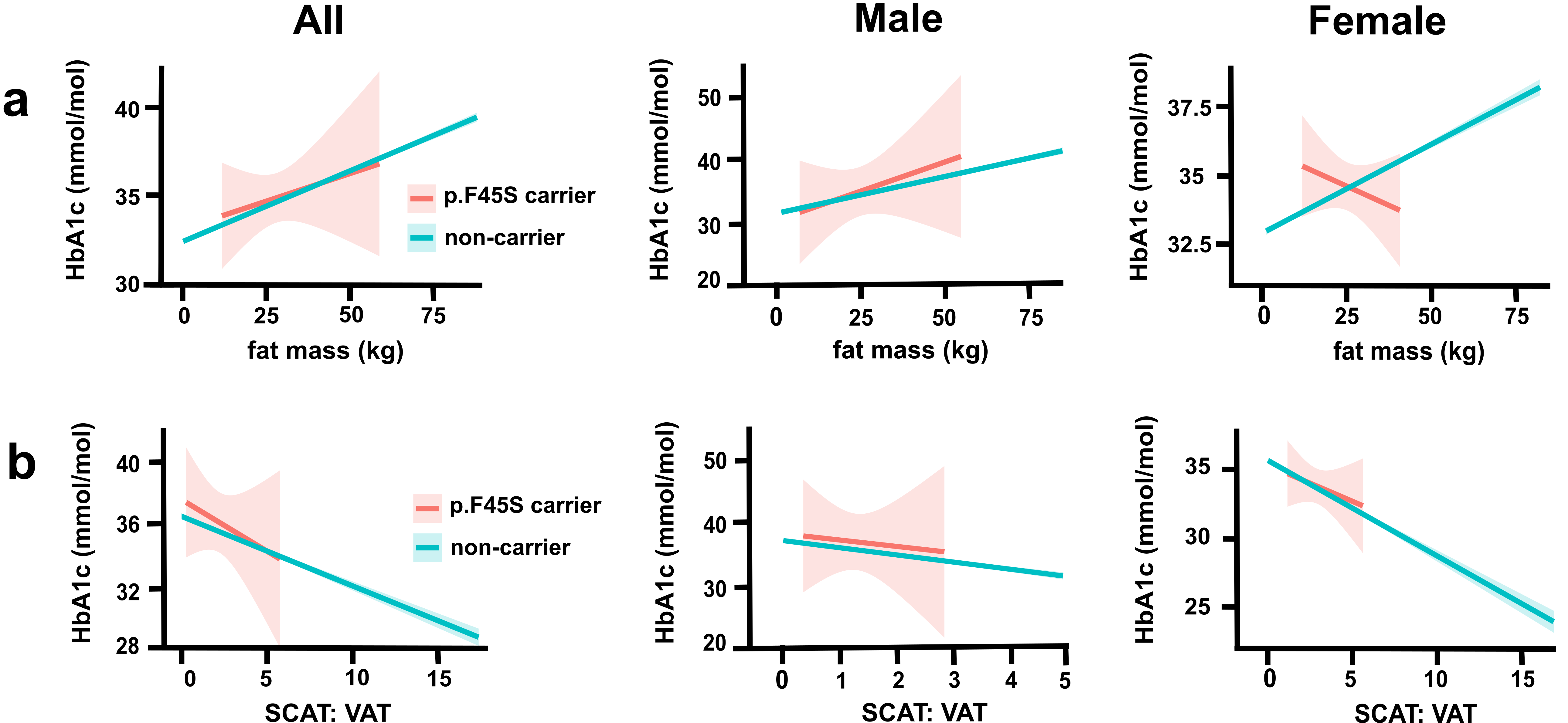

Supplement: Supplementary file 1 — FIGURE S1. MC3R p.F45S associations of HbA1c with fat mass variables. Associations were plotted between (A) total fat mass and glycated haemoglobin levels and (B) subcutaneous‐to‐visceral fat mass ratio and glycated haemoglobin levels, to compare carriers and non‐carriers of MC3R p.F45S within and between genetic sexes. Plots depict mean ± SE, and colours indicate carrier status: heterozygote for p.F45S (orange); non‐carrier (green). Differences were assessed using a linear mixed model, but there were no significant differences. N, beta values, standard errors, p values, and correlation data are listed in Table S6. HbA1c, glycated haemoglobin; SCAT, subcutaneous adipose tissue; VAT, visceral adipose tissue. [file JNE-38-e70214-s002.png]
